# Evaluation of Internal Fit in Custom-Made Posts and Cores Fabricated with Fully Digital Versus Conventional Techniques

**DOI:** 10.3390/jfb15120389

**Published:** 2024-12-22

**Authors:** Eric Jensen, Shariel Sayardoust

**Affiliations:** 1 Centre for Oral Rehabilitation, Linköping, County Council of Östergötland, 581 86 Linköping, Sweden; eric.jensen@regionostergotland.se; 2 Department of Biomedical and Clinical Sciences, Linköping University, 581 83 Linköping, Sweden

**Keywords:** dental impression technique, digital dental technology, vinyl polysiloxane, dental fit, computer-aided design, post-and-core technique

## Abstract

Objective: This study aimed to assess and compare the internal fit of custom-made posts and cores fabricated using digital impressions (DI) and conventional vinyl polysiloxane (VPS) impressions in restorative dentistry. Materials and Methods: A typodont tooth model, simulating the anatomy of the root canal of a central incisor, was utilized for the study. Two groups were formed, Group A and Group B, and each group provided a total of 18 impressions of two types: DIs and VPS impressions. In Group A, posts and cores (PCs) were fabricated using Selective Laser Melting (SLM) from the DIs. Meanwhile, in Group B, conventionally cast (CC) PCs were created from the VPS impressions. Silicone replicas of the internal surfaces were produced, and measurements were made at seven different points for each group. A statistical analysis was performed to assess the differences in internal fit between the two impression techniques. Results: The results revealed a statistically significant difference in mean internal fit between Group A (DI and SLM) and Group B (VPS and CC), with Group A exhibiting a mean internal fit of 182.6 µm and Group B showing a mean of 205.9 µm. While both groups demonstrated considerable variability in internal fit measurements, the digital impression technique showed promise for achieving superior internal fit, with a significantly greater fit for measuring points on sides and the most apical part of the post for Group A. Variations were observed across different measuring points, emphasizing the impact of impression technique on specific regions within the tooth. Conclusion: This study contributes to the growing body of knowledge in digital dentistry by highlighting the potential benefits of DIs in achieving a superior internal fit for custom-made PCs. Clinicians may consider the advantages of digital techniques to enhance the precision of their restorative procedures, although further research is warranted to evaluate the clinical impact of these findings.

## 1. Introduction

The principle of rebuilding a tooth with a post and core to enhance its retention has long been a common treatment option in dentistry. Since Pierre Fauchard’s pioneering description of techniques for restoring teeth with metal posts and cores in 1728, this approach has become a fundamental aspect of dental practice [1]. Over the years, dentists have employed a variety of methods, including individually formed posts and cores (PCs) using indirect cast techniques, as well as the use of different prefabricated posts for direct applications. These techniques have evolved into routine practices among dental professionals. Clinical follow-up studies have consistently demonstrated survival rates ranging from 50% to 100% over periods of 6 months to 10 years for restored teeth with different types of PCs [2]. These findings underscore the pivotal roles played by meticulous planning, appropriate materials, precise execution, and sound techniques in achieving successful post and core restorations.

One of the prevailing impression techniques employed to reproduce the inner tooth anatomy for indirect post and core fabrication is the use of vinyl polysiloxane (VPS) impression material. Following this step, a stone model is generated, serving as the basis for wax build-up. Upon approval of the wax build-up, the post and core are conventionally cast in metal. However, recent decades have witnessed a transformation in the field of dentistry with the gradual development of digital impression (DI) techniques, which have evolved since the 1970s [3]. Emerging research suggests that the marginal and internal fit of crowns fabricated using contemporary intraoral impression scanning devices are on par with or even superior to those achieved through conventional VPS impressions [4,5,6,7]. Recent studies have demonstrated that PC restorations fabricated using DIs can achieve outcomes comparable to or superior to those produced with conventional VPS impressions. Additionally, these techniques have been shown to result in clinically acceptable fits for restorations [8,9].

While advancements in digital impression techniques have shown promising results, discrepancies remain in the literature regarding the accuracy and adaptation of digitally fabricated PC restorations compared to conventional techniques. For example, studies by Leven et al. [10] and Vogler et al. [11] highlight the superior fit achieved with digital workflows, whereas others, such as Perucelli et al. [9] and Jafarian et al. [8], report better adaptation and retention in posts and cores fabricated using conventional or hybrid workflows. These conflicting findings underscore the need for further research to clarify the comparative accuracy and clinical implications of these techniques. This study aims to address this gap by evaluating the internal fit of post-and-core restorations fabricated using fully digital workflows versus traditional VPS impressions and casting techniques. Given the current clinical availability of DI techniques, a pressing need arises for a thorough comparative evaluation of these two approaches. In the fabrication of custom metal posts and cores from digital impressions, modern methods such as milling or Selective Laser Melting (SLM) have supplanted conventional casting (CC) techniques. Milling and Selective Laser Melting (SLM) are modern techniques used for fabricating custom-made dental restorations. Milling involves subtractive manufacturing, where a solid block of material (e.g., metal or ceramic) is precisely shaped using computer-aided machinery. This method offers high precision and smooth surface finishes but often results in significant material waste and limited design complexity. In contrast, SLM is an additive manufacturing technique that builds restorations layer by layer using a laser to melt powdered metal. SLM allows for greater design flexibility and less material waste compared to milling. However, it may result in rougher surface textures that require additional post-processing. Both techniques represent advancements over CC, which involves creating restorations using wax patterns and molten metal, but each has unique benefits and limitations that influence their clinical applications. Therefore, this study seeks to fabricate posts and cores from DI using SLM, while the VPS group will adhere to the CC procedures. This differentiation in fabrication methods will allow us to assess the comparative accuracy and efficacy of these techniques, with the DI group representing the contemporary fully digital workflow of PC fabrication and the VPS group simulating a traditional approach.

In an era of dynamic advancements in dental technology, understanding the nuances and implications of these fabrication techniques is pivotal to continually enhancing the quality of dental restorations. This study aims to contribute valuable insights into the evolving landscape of post-and-core restorations, shedding light on the potential advantages and limitations of digital impressions and modern fabrication methods in the context of contemporary dental practice.

### Objective

The primary objective of this investigation is to assess the internal fit of custom-made PC restorations fabricated using a fully digital workflow (digital impressions combined with Selective Laser Melting) compared to those produced using a conventional workflow (vinyl polysiloxane impressions combined with traditional casting techniques).

The hypothesis was that PCs made from modern, digital methods should perform equally as good regarding fit as conventionally made PCs.

## 2. Materials and Methods

A typodont tooth (AG-3 ZPUR, Frasaco Gmbh, Tettnang, Germany) with a root canal anatomy closely resembling that of the left central incisor was meticulously prepared for the fabrication of a custom-made metal PC by an experienced dentist. The coronal tooth was vertically reduced and horizontally planed, maintaining a 2 mm circumferential ferrule to ensure structural integrity. The preparation was 12 mm deep and 2 mm wide, tapering in the apical direction. Root filling material was removed using Gates-Glidden reamers, and the canal was further widened using a tapered Dentatus root reamer to achieve a uniform taper.

This investigation employed two distinct impression methods: the conventional approach using VPS impression material (Provil Light, Monophase, Heraeus Gmbh, Hanau, Germany) reinforced with an impression post (Burnout post, Directa AB, Väsby, Sweden) within a disposable styrene tray (Tray aways, Bosworth Co., East Providence, RI, USA). Both the tray and impression post were pre-treated with VPS impression adhesive (Universal adhesive, Heraeus Gmbh, Hanau, Germany). Additionally, DIs were obtained using the Trios 3 intraoral scanner (3 Shape). The digital scanning process followed a standardized sequence: occlusal views were captured first, followed by buccal, lingual, and detailed fill-ins. All impressions were inspected for flaws, such as the penetration of the impression post through the impression material; no such flaws were observed. In total, eighteen impressions of each type were generated, alternating between DI and VPS techniques to minimize bias and identify potential systematic errors. The visual inspection of the typodont tooth and impressions, along with scanning inspections, confirmed no significant remnants of VPS material.

Custom-made PCs were additively manufactured with SLM techniques from a powder bed of cobalt chromium alloy grains (Remanium Star CL, Dentauru Gmbh, Ispringen, Germany) fused in layers under focused heat, planned and derived from CAD (Computer-Aided Design). For the digital impressions, the PCs were directly designed on the digital impressions with a preset digital spacer of 60 µm. Spacers were applied in both groups to standardize fit assessment. No further adjustments were made for the SLM posts. For the VPS impressions, a type IV stone model (Prima-Rock, Whip Mix, Louisville, KY, USA) was fabricated, followed by wax build-up (K2 Exact, Bredent Gmbh, Senden, Germany). The wax pattern was invested (Magma Speed, Bracon Ltd., London, UK), and the post and core were cast in cobalt chromium alloy (Biodur-NP, Bracon Ltd., London, UK). Following casting, the PCs underwent divestment and were subjected to sandblasting with 120 µm of grit AlO_3_ powder. Minimal adjustments were performed by the dental technician to ensure a proper fit on the plaster working models.

Prior to creating the silicone replicas, the inner surface of the typodont model was treated with Microfilm (Kerr, Brea) to prevent tearing when removing the post. The silicone replica technique, a validated and widely used method for assessing the internal fit of prosthodontic restorations in vitro, was conducted in collaboration with Professor A. Falk at Malmö University, where the silicone replica technique has been extensively validated and used in previous research. The technique remains a reliable standard for evaluating internal fit in restorative dentistry [12,13]. The silicone replicas were crafted from the posts and cores on the typodont model using two materials:

1. Fit Checker Blue (GC Dental, Mumbai, India): applied to the record gap between the post and the canal.
2. Aquasil Ultra+ (Sirona Dental Systems Gmbh, Mumbai, India): injected to fill out and stabilize the fit checker layer, when post was removed.

**Figure 1:**
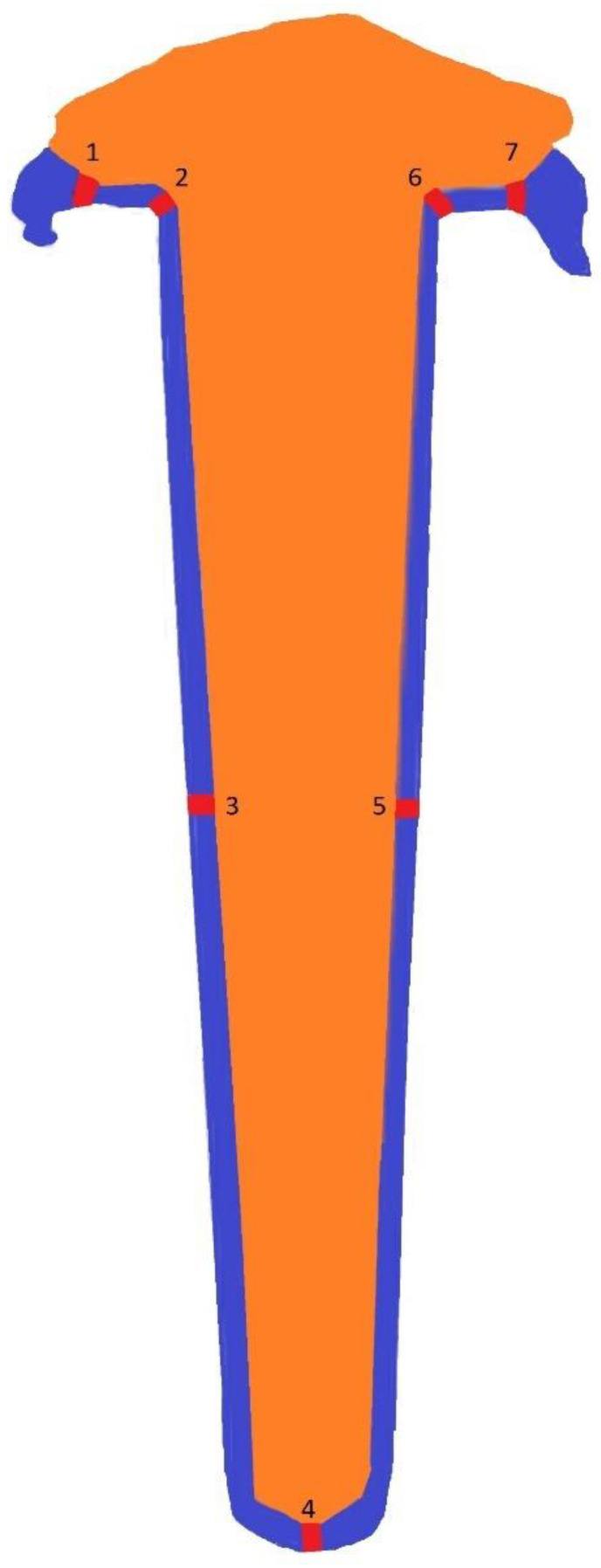
Showing the distribution of measuring points (1 is buccal and 7 is lingual in Table 1 and 1 is mesial and 7 is distal in Table 2).

Each silicone replica was sectioned vertically into four quadrants (mesial–distal and buccolingual) using a razor. The PC itself was not sectioned; instead, its space was filled with silicone impression material to ensure accurate measurements of the internal fit. Measurements were taken at seven distinct points for both buccolingual and mesiodistal sections to evaluate fit at critical locations along the restoration (Figure 1):

1. Point 1: Marginal area.
2. Point 2: Inner angle at the margin.
3. Point 3: A 6 mm depth from the margin on one side.
4. Point 4: Apical gap.
5. Point 5: A 6 mm depth from the margin on the opposite side.
6. Point 6: Inner angle at the opposite margin.
7. Point 7: Lingual or distal margin (depending on section type).

**Table 1:** Buccolingual sections. Mean internal gap measurements (µm) at seven distinct buccolingual measuring points for Group A (fully digital workflow) and Group B (conventional workflow). The mean and standard deviation (SD) of internal gap measurements are presented for each measuring point. Statistical significance was determined using independent samples *t*-tests, with a *p*-value < 0.05 considered significant.

Group A	1	2	3	4	5	6	7
Mean	226.8889	233.7222	105.4444	170.2222	117.1667	191.3333	202.7222
SD	64.25857	71.14344	30.2387	61.29538	49.66269	54.00218	93.73041
Group B	1	2	3	4	5	6	7
Mean	277.1667	205.0556	87.94444	255.2778	136.0556	194	258.3889
SD	93.2178	90.84795	45.86614	103.8289	72.83299	65.99554	93.80778
*p*-value BL	0.068134	0.299309	0.185467	0.005118	0.369706	0.895233	0.083854

**Table 2:** Mesiodistal sections. Mean internal gap measurements (µm) at seven distinct mesiodistal measuring points for Group A (fully digital workflow) and Group B (conventional workflow). The mean and standard deviation (SD) of internal gap measurements are presented for each measuring point. Statistical significance was determined using independent samples *t*-tests, with a *p*-value < 0.05 considered significant.

Group A	1	2	3	4	5	6	7
Mean	223.5	200.0556	108.2778	185.6667	129.9444	245.5556	216.0556
SD	44.99967	35.53204	41.87068	58.20956	54.02339	61.1317	93.93958
Group B							
Mean	260.0556	219.7778	99.5	275.8333	109.2222	200.4444	303.5
SD	84.33231	88.93326	75.71327	98.89225	86.15278	68.36599	86.51368
*p*-value MD	0.11393	0.388405	0.669597	0.002079	0.39334	0.044469	0.006414

These points were selected to capture variations in fit along the apical–coronal and mesial–distal axes of the post and core. The alternation of digital and conventional impressions ensured that any potential degradation in precision due to successive impressions could be identified, minimizing systematic errors and ensuring the reliability of the methodology. Measurements were made using the measuring tool through the lens with 10× magnification light microscopy (Leica WILD M7A, Leica Gmbh, Lindau, Germany).

**Figure 2:**
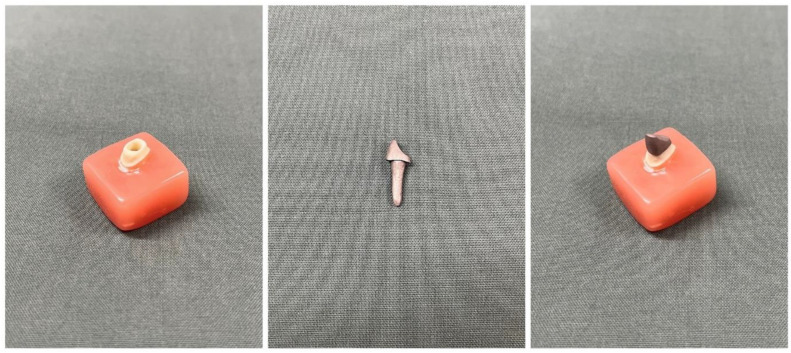
A photograph of the master model and one of the post and core specimens has been included to visually represent the experimental setup and fabrication process.

The collected data were subjected to statistical analysis using SPSS v.29 (IBM, New York, NY, USA). Descriptive statistics summarized the mean internal fit values for each group at the specified measuring points. An independent samples *t*-test was used to compare the means of the two groups (Group A and Group B). A *p*-value of less than 0.05 was considered statistically significant, indicating whether observed differences in mean internal fit were likely due to the manufacturing method rather than random variation. A photograph of the master model and one of the post and core specimens has been included to visually represent the experimental setup and fabrication process (Figure 2).

## 3. Results

### Comparison of Mean Internal Fit

This study investigated the differences in mean internal fit between digitally made posts and cores (Group A) and conventionally made counterparts (Group B). Measurements were taken at seven distinct points, ranging from apical to coronal sections. The mean internal fit values for Group B varied between 99.500 µm and 303.500 µm, while for Group A, the range was slightly narrower, from 108.278 µm to 245.556 µm (Table 1).

**Figure 3:**
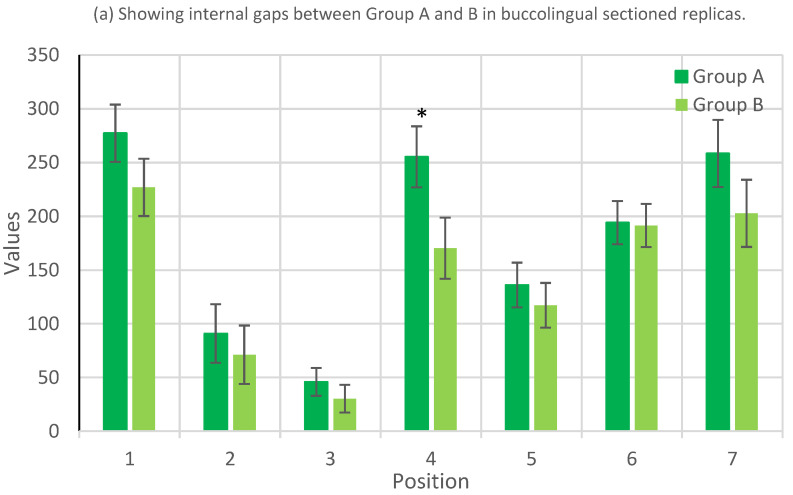

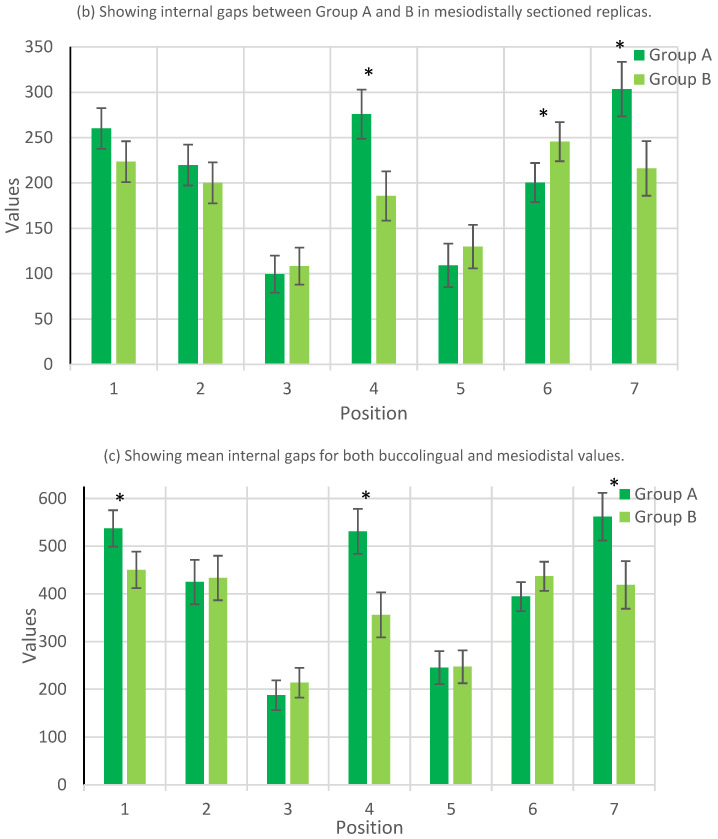
(**a**) Showing internal gaps between Group A and B in bucco-lingual sectioned replicas. (**b**) Showing internal gaps between Group A and B in mesiodistally sectioned replicas. (**c**) Showing mean internal gaps for both buccolingual and mesiodistal values. (Statistically significant differences are marked *, *p* < 0.05).

Significant differences were observed at specific measuring points. The most pronounced discrepancy was noted at Measuring Point 4, where Group B exhibited a mean internal fit of 275.833 µm compared to Group A’s 185.667 µm, with a *p*-value of 0.002, indicating a statistically significant difference. Similarly, Measuring Points 6 and 7 also showed statistically significant differences with *p*-values of 0.044 and 0.006, respectively (Figure 3 and Table 3).

The statistical significance of the differences between the two groups was assessed using *p*-values derived from a *t*-test. Measuring Points 1, 2, 3, and 5 did not show statistically significant differences, with *p*-values ranging from 0.114 to 0.670. In contrast, Measuring Points 4, 6, and 7 demonstrated statistically significant differences (*p* < 0.05), indicating a notable variance in the internal fit between the digitally and conventionally made PCs at these specific locations (Figure 3 and Table 3).

These results underscore the variability in internal fit between different manufacturing techniques for PCs. Notably, the apical section (Measuring Point 4) and the coronal sections (Measuring Points 6 and 7) displayed the most significant discrepancies. This finding suggests potential areas for improvement in manufacturing processes, particularly in achieving consistency in internal fit across various sections of posts and cores.

## 4. Discussion

The present study aimed to evaluate the internal fit of custom-made posts and cores fabricated using DIs compared to those produced through conventional VPS impressions. The significance of this investigation lies in the evolution of digital dentistry techniques and their potential impact on the accuracy and precision of restorative procedures.

Comparison of internal fit: The results of our study revealed a difference in mean internal fit between Group A (DI) and Group B (VPS), with Group A exhibiting a mean internal fit of 182.6 µm and Group B showing a mean of 205.9 µm. These findings suggest that the digital impression technique may offer advantages in achieving a superior internal fit compared to conventional VPS impressions. The smaller mean internal fit observed in Group A implies a potentially better adaptation of digitally fabricated posts and cores to the tooth anatomy. Similar studies have found resembling results, where digital methods show a better fit than conventional impressions [10,11].

Variability in internal fit: Both Group A and Group B demonstrated considerable variability in internal fit measurements, with a wide range of values observed. This variability underscores the importance of precise planning, materials, and execution in post-and-core treatments. The lowest and highest values of misfit were found to be quite divergent in both groups, emphasizing the need for meticulous attention to detail in restorative dentistry. The variability observed between different measurement points in this study may be attributed to several factors. One key factor is the preparation depth, as deeper regions of the canal may pose challenges in capturing accurate impressions due to limited accessibility and increased potential for distortion [8]. Additionally, the type of scanner used for digital impressions can significantly influence accuracy. While the Trios 3 intraoral scanner used in this study is known for its high resolution, factors such as angulation, the presence of adjacent teeth, and the reflective properties of the canal walls may have impacted the precision of the scans. These factors, combined with inherent limitations in both digital and conventional techniques, likely contributed to the variability observed in the internal fit measurements. Future studies should further investigate how preparation design and scanning parameters affect fit consistency.

Contrary to these findings, a study by Hendi et al. concluded that conventionally made posts and cores have superior fit and resistance compared to those produced fully digitally [14]. However, the fully digitally made PCs in these cases were produced through milling, not SLM like in this study. This suggests that the production method significantly influences the fit of a digitally made PC.

Influence of measuring points: The analysis of internal fit measurements across different measuring points within the mesiodistal sections provided valuable insights. In Group A, the highest mean values were observed in the inner angle of the distal part and the mesiodistal sectioned replica (Measuring Point 6), whereas in Group B, the most substantial mean value was found at the most apical part of the mesiodistal sectioned replicas (Measuring Point 4). These variations suggest that the choice of impression technique may impact the fit of specific regions within the tooth indicate that measurement variations depend on several factors: the depth and width of the preparation, the type of intraoral scanner used, and the presence of adjacent teeth [15].

Clinical implications: The clinical implications of our findings are noteworthy. The superior mean internal fit observed in digitally fabricated posts and cores may have implications for the longevity and success of dental restorations. Improved adaptation to tooth anatomy may enhance retention and stability, ultimately contributing to the long-term health of the restored tooth. The clinical implications of internal adaptation in post-and-core restorations are significant. Studies have shown that a superior internal fit improves the mechanical stability and retention of restorations, ultimately contributing to their longevity [2,16]. Furthermore, a better fit reduces the risk of microleakage, which is a key factor in preventing secondary caries and other complications [6]. These findings suggest that the improved fit observed with digitally fabricated posts and cores in this study may positively impact clinical outcomes, supporting the adoption of digital workflows in restorative dentistry.

Additionally, as concluded in a study by Libonati et al., digital techniques often offer a faster method with fewer working steps, both in the lab and clinic. This can ultimately lead to increased productivity and fewer procedural errors [16].

Limitations and future directions: It is important to acknowledge the limitations of this study. The evaluation of internal fit was limited to specific measuring points, and other factors such as occlusal forces and clinical performance were not assessed. Future research could explore the relationship between internal fit and clinical outcomes over an extended follow-up period. While digital workflows offer numerous advantages, such as enhanced precision and reduced procedural steps, they are not without limitations. One significant challenge is the learning curve associated with mastering intraoral scanning and CAD software. Operator skill and experience can significantly influence the accuracy of digital impressions, as subtle errors in scanning technique may lead to distortion or incomplete data capture [6]. Additionally, the initial cost of acquiring digital equipment, such as intraoral scanners and CAD/CAM systems, may pose a barrier for smaller practices. Maintenance and software updates are other practical considerations that can add to the overall cost. These factors highlight the importance of proper training and ongoing education for clinicians transitioning to digital workflows to ensure consistent and reliable results. Future studies should explore how these practical considerations impact clinical outcomes and adoption rates.

One other limitation of this study is the lack of 3D imaging or volumetric analysis. Although we initially explored high-definition scanning methods, the narrow and parallel design of the post space made accurate scanning unfeasible. Consequently, we utilized the validated silicone replica technique, which ensures comparability with previous research. Future studies should incorporate advanced imaging techniques such as CT or 3D scanning to provide a more comprehensive evaluation of internal fit.

The CAD software predefined the cement spacing as 60 µm, which was applied uniformly across all samples. However, exact error margins for the measuring process were not quantified in this study, representing another limitation. Future research should focus on quantifying measurement error and accuracy using high-precision metrology tools.

Additionally, the limited sample size in this study represents a potential limitation, as it may affect the generalizability of the findings. Although the controlled experimental setup allowed for detailed comparisons between digital and conventional workflows, larger-scale studies involving a broader range of clinical conditions and tooth types are needed to confirm these results. Additionally, future research could include clinical trials to assess the performance of these techniques under real-world conditions. Expanding the sample size and scope of the study would provide more robust data to support the adoption of digital workflows in restorative dentistry.

Further research should focus on evaluating differences in production technique in milling versus SLM.

## 5. Conclusions

In conclusion, our study suggests that posts and cores fabricated with the SLM technique, based on digital impressions, may offer a superior internal fit compared to conventionally cast counterparts using VPS impressions. However, both methods exhibit variability in fit measurements, emphasizing the importance of precision in restorative dentistry. These findings contribute to the growing body of knowledge in digital dentistry and provide valuable insights for clinicians seeking to optimize their restorative procedures. Further research is needed to validate the clinical impact of improved internal fit on the long-term success of dental restorations.

## Figures and Tables

**Table 3:** Comparison of mean internal gap measurements (µm) between Group A (fully digital workflow) and Group B (conventional workflow) across buccolingual and mesiodistal sections. (a) Showing internal gaps between Group A and B in buccolingual sectioned replicas (b) Showing internal gaps between Group A and B in mesiodistally sectioned replicas. (c) Showing mean internal gaps for both buccolingual and mesiodistal values. (Statistically significant differences are marked *, *p* < 0.05).

**(a)**	**Position**	**A**	**B**	***p*** **-value**	**SEM**
	1	277.17	226.89	0.084	26.69
	2	90.85	71.14	0.299	27.20
	3	45.87	30.24	0.185	12.95
	4	255.28	170.22	0.005 *	28.42
	5	136.06	117.17	0.370	20.78
	6	194.00	191.33	0.895	20.10
	7	258.39	202.72	0.084	31.26
**(b)**	**Position**	**A**	**B**	***p*** **-value**	**SEM**
	1	260.06	223.50	0.114	22.53
	2	219.78	200.06	0.388	22.57
	3	99.50	108.28	0.670	20.39
	4	275.83	185.67	0.002 *	27.05
	5	109.22	129.94	0.393	23.97
	6	200.44	245.56	0.044 *	21.62
	7	303.50	216.06	0.006 *	30.10
**(c)**	**Position**	**A**	**B**	***p*** **-value**	**SEM**
	1	537.22	450.39	0.029 *	38.15
	2	424.83	433.28	0.839	46.63
	3	187.44	213.72	0.404	31.10
	4	531.11	355.89	<0.001 *	47.25
	5	245.28	247.11	0.958	34.53
	6	394.44	436.89	0.172	30.40
	7	561.89	418.78	0.007 *	50.01

## Data Availability

The original contributions presented in the study are included in the article, further inquiries can be directed to the corresponding author.
